# Prevalence and prevalence trends of transfusion transmissible infections among blood donors at four chinese regional blood centers between 2000 and 2010

**DOI:** 10.1186/1479-5876-10-176

**Published:** 2012-08-28

**Authors:** Changqing Li, Xiaopu Xiao, Huimin Yin, Miao He, Jianping Li, Yudong Dai, Yongshui Fu, Jianmin Ge, Yonglin Yang, Yan Luan, Changzhou Lin, Hongxiang Zhao, Wuping Li

**Affiliations:** 1 Institute of Blood Transfusion, Chinese Academy of Medical Sciences and Peking Union Medical College, Hua Cai Road 26 Hao, Dong San Huan Road Er Duan, Chengdu, Sichuang, 610052, China; 2 Department of Biology, Faculty of Pure and Applied Science, Acadia University, Wolfville, Canada; 3 Liao Ning Blood Center, Shenyang, China; 4 Nan Jing Blood Center, Nanjing, China; 5 Guang Zhou Blood Center, Guangdong, China; 6 Yan Cheng Blood Center, Nanjing, China; 7 Institute of Pathogen Biology, Chinese Academy of Medical Sciences and Peking Union Medical College, Beijing, China

**Keywords:** Transfusion, Prevalence, HIV, HBV, HCV, Syphilis

## Abstract

**Background:**

In China, high prevalence of HBV and HCV parallels with the growing epidemic of syphilis and HIV in the general population poses a great threat to blood safety. This study investigated the prevalence of serologic markers for transfusion transmissible infections (TTIs) among four Chinese blood centers.

**Methods:**

We examined whole blood donations collected from January 2000 through December 2010 at four Chinese blood centers. Post-donation testing of TTIs (HIV, HBV, HCV and syphilis) were conducted using two different enzyme-linked immunosorbent assay kits for each seromarker. The prevalence of serologic markers for TTIs (%) was calculated and additional analysis was conducted to examine donor characteristics associated with positive TTIs serology.

**Results:**

Of the 4,366,283 donations, 60% were from first-time donors and 40% were from repeated donors. The overall prevalence of HIV, HBsAg, HCV and syphilis was 0.08%, 0.86%, 0.51% and 0.47%, respectively. The prevalence profile of TTIs varied among different blood centers and appeared at relatively high levels. Overall, the prevalence of HBsAg and HCV demonstrated a decline trend among four blood centers, while the prevalence of HIV and syphilis displayed three different trends: constantly steady, continually increasing and declining among different centers.

**Conclusions:**

This study reflects the risk of TTIs has been greatly reduced in China, but blood transfusion remains an ongoing risk factor for the spread of blood-borne infections, and further work and improvements are needed to strengthen both safety and availability of blood in China.

## Background

Human blood is a major source of diverse medical products that are used for the prevention and treatment of various life-threatening diseases. However, blood transfusion has been subjected to contamination with different human pathogens that may induce a wide variety of risk, especially transfusion-transmissible infections (TTIs) such as HIV, HBV, HCV and syphilis. Over the past three decades, the risk of TTIs has been dramatically reduced by the introduction of routine donor laboratory screening of blood-borne pathogens [1,2]. In the EU and the United States, due to continuous implementation and improvement of more sensitive serologic methods and nucleic acid amplification test (NAT), the residual risk of viral transmission decreased in 2000 to less than 1: 250,000 for hepatitis C virus (HCV) and 1: 1.3 M for HIV [1,3]. By contrast, TTIs still pose a great threat to blood safety.

In China, whole blood units for clinical use are collected at blood centers/banks. There are more than 452 blood centers/banks at three levels: provincial (32), regional (321), and county (99). Local government health offices oversee the operation of Chinese blood centers/banks. Since the Blood Donation Law came into effect in 1998, many changes have been made in the field of blood banking. Blood collection has been successfully shifted from paid and employer-organized donations to voluntary donation. As a result, voluntary donations have increased from 5.5% (50,000 donations) in 1998 to 99% (12,320,000 donations) in 2011. Overall blood collection has increased from fewer than 1000 tons to 4164 tons. Although blood collection volume has increased dramatically, the increase in the blood supply has not kept pace with the increasing clinical demand for blood[4]. The blood donation rate is only 9 ‰ of the whole population, much lower than that the WHO demands: 10–30 ‰. In the area of donor screening in China, a standardized national donor screening policy has been implemented. It requires that (1) all potential donors undergo a screening process to meet the donor eligibility requirements before donating; (2) after passing the predonation screening process, blood units will be collected and undergo two rounds of routine serological testing for HBsAg, ALT, hepatitis C virus (HCV) antibodies, human immunodeficiency virus (HIV) antibodies, and syphilis antibodies by two different reagents which are imported as well as domestic testing kits approved and licensed by the Chinese State Food and Drug Administration.

Although the prevalence of these diseases in a general population of selective participants has been addressed, there are limited epidemiological data on TTIs in blood donors in China. As the infection increasingly spreads further into the general population and the number of HIV cases keeps growing rapidly in China, the risk of transfusion-transmitted HIV poses a conspicuous threat to blood safety [5,6]. Previous studies have demonstrated that the prevalence of HBV and HCV is quite high in China, approximately 9.8% of the Chinese population tested positive for hepatitis B virus surface antigen (HBsAg) before the introduction of the HBV vaccination program in 1992. Fortunately, the HBsAg carrier rate decreased to 7.2% among the general population in 2006 [7,8]. The nationwide prevalence of HCV infection was estimated to be 3.2% in 1992. The regional data indicated that the prevalence of HCV rose to 12.87% of blood donors before 1998 and dramatically decreased to 1.71% later on [9]. However, epidemiological studies of HCV are limited compared to those of HBV [10,11]. During the past 20 years, syphilis has made resurgence in China. In 1993, the reported overall rate of cases of syphilis was 0.2 cases per 100,000 persons, whereas primary and secondary syphilis alone represented 5.7 cases per 100,000 persons in 2005. Furthermore, the rate of congenital syphilis increased sharply from 1991 to 2005, from 0.01 cases per 100,000 live births to 19.68 cases per 100,000 live births, with an average yearly rise of 71.9% [12]. One study demonstrated that there was a significant growth in syphilis serologic markers among first-time donors from 2008 to 2010 [13]. Although a number of studies have monitored the epidemiology of HIV, HBV, HCV and syphilis in China’s general population and its high-risk population, data revealing the epidemiology from blood donor groups are significantly limited.

In the present study, we assessed the prevalence of TTIs among voluntary blood donors in four blood centers from 2000 to 2010. A retrospective review of donors’ record covering the period was carried out. All samples were screened for HIV, HBsAg, HCV, syphilis. The safety of the blood supply can be estimated by monitoring the prevalence of these pathogens in blood donations, and a reliable index for policy making can be produced.

## Methods

### Participants and principle of the study

**Figure 1:**
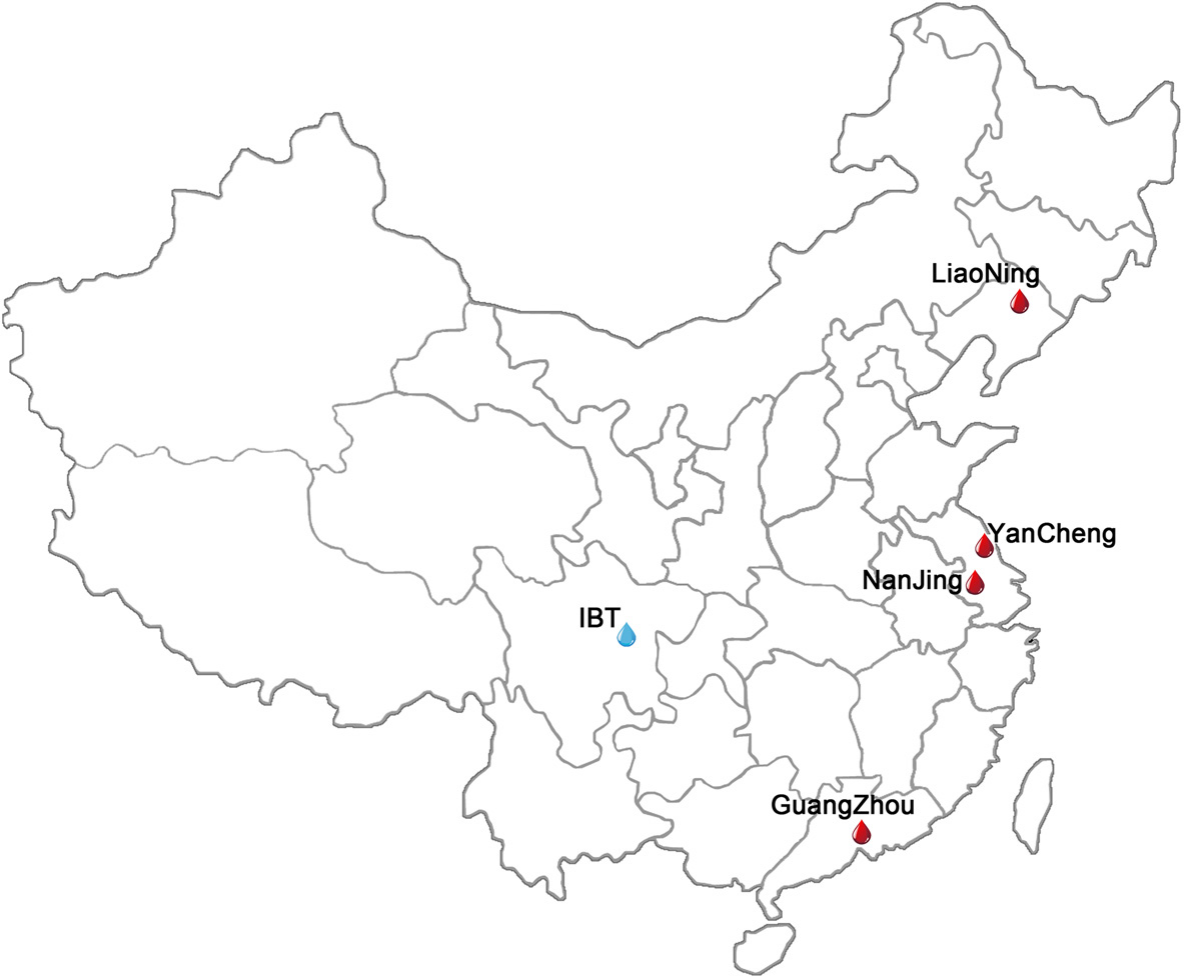
**Locations of study sites in China.** IBT = Institute of Blood Transfusion.

The Institute of Blood Transfusion at Chengdu (China), collaborated with four Chinese regional blood centers in Guangzhou, Nanjing, Liaoning and Yancheng. These four centers were the participants of this study. Figure 1 shows the geographic distribution of the four blood centers and the Institute of Blood Transfusion. The four blood centers were selected for their geographic and economic level: Guangzhou, located in Southeast China, represents an economically developed region; Liaoning, located in Northeast China, represents an economically developing region; Nanjing and Yancheng, located in mid-East China, also represent an economically developed region. The first three are provincial capital city level blood centers and Yancheng is a blood center serving second-tier (smaller) cities. The study population consists of all blood donors who donated at one of the four participating blood centers or at one of their mobile blood collection vehicles between January 1, 2000 and December 31, 2010. All blood donations were collected and screened for the serological makers for HIV, HBV, HCV and syphilis, and the seroprevalence of these pathogens in blood donors was evaluated to investigate the blood safety in China.

### Pre-donation screening

Due to the high prevalence of HBV and HCV in China’s general population and exclude potential window period infection, all blood donors were required to pass a routine pre-donation screening process, including a health history questionnaire screening, pre-donation rapid testing, and a brief physical examination. The health history questionnaire has minor variations across the participating blood centers, but all versions contained the required screening fields mandated by the Chinese Ministry of Health. Pre-donation rapid testing procedures vary across blood centers, but all tested for hepatitis B virus surface antigen (HBsAg), ABO blood type, and hemoglobin. Alanine aminotransferase (ALT) screening was also being performed at selected centers. Liaoning blood center began HBsAg predonation rapid testing in May 2001; all the other blood centers began this test before 2000. Risk behavior detected in the health history questionnaire, an abnormal result in pre-donation rapid testing or failure to pass the physical examination (e.g., body weight lower than 45 kg) resulted in the donor’s deferral. Donors who meet the donor eligibility requirements proceeded to donate blood.

### TTIs testing

After a donor passed the pre-donation screening, a blood unit was collected. All donor samples underwent two rounds of enzyme immunoassay (EIA) testing with two different reagents at the blood centers for the following routine tests: ALT, anti-HIV-1 and −2, anti-HCV, HBsAg, and syphilis antibody. The two different reagents used were imported and domestic testing kits. All kits were approved and licensed by the Chinese State Food and Drug Administration. All reagents used in donor screening are varied by blood centers, and are listed in Table 1. A duplicative test would be performed if a sample was reactive to one of the rounds of the post-donation screening tests, using the kit whose result had been nonreactive. If either test was reactive, the result was considered to be reactive. The screening “combo” result, which is the final screening result, was determined on duplicative testing. A positive combo result would defer a donor and that involved donated unit would be discarded. Reactive anti-HIV test results are required to be confirmed at local public health station HIV reference laboratories certified by the Ministry of Health. Staffs from local public-health stations undertake notification and consultation of donors. Confirmatory testing for anti-HCV, HBsAg, and syphilis antibodies is not routinely done because of the large number of donors reactive with anti-HCV, HBsAg, and syphilis antibodies at screenings as well as the high cost of confirmatory testing. There is no national standardized programme to notify donors about positive test results—except for HIV—but all donors with positive test results are informed by telephone and email, or donors learn it by themselves using the website of the blood center that is selected by the blood donor before donation. In order warrant the blood supply in China, we advocate the disclosure of results of TTIs to blood donors by a well-defined notification processes to avoid side-effect resulting from unguided disclosure. For this analysis, we used TTI screening test results to calculate TTI prevalence rates in Whole blood (WB) donations. The prevalence rate was defined as the number of all screening positives, excluding ALT, divided by the overall number of WB donations.

**Table 1:** Reagents and company used in donor screening

**Kit Name**	**Company**
Diagnostic Kit for Antibody to HIV (Sandwich ELISA)	Beijing WANTAI Biological Pharmacy Enterprise Co., Ltd.Zhuhai Livzon Diagnostics INC.InTec Products, INC.(XIAMEN)BIO-RAD
Diagnostic Kit for Antibody to HCV (ELISA)	Beijing WANTAI Biological Pharmacy Enterprise Co., Ltd.Zhuhai Livzon Diagnostics INC.InTec Products, INC.(XIAMEN)Abbott Molecular
Diagnostic Kit for Antibody to HBV Surface Antigen(ELISA)	Beijing WANTAI Biological Pharmacy Enterprise Co., Ltd.Shanghai Kehua BIO-ENGINEERING CO., LTDInTec Products, INC.(XIAMEN)bioMérieux Clinical Diagnostics
Diagnostic Kit for Antibody to Treponema Pallidum(ELISA)	Beijing BGI-GBI Biotech Co., Ltd.Beijing WANTAI Biological Pharmacy Enterprise Co., LtdInTec Products, INC.(XIAMEN)

### Statistical analysis

Statistical analyses were conducted using SPSS 11.5 statistics software. The Chi-square test was applied to assess the association between the categorical variants. A P-value of < 0.05 was used as the cut-off level for significance.

## Results

### Increase in whole blood collection

**Figure 2:**
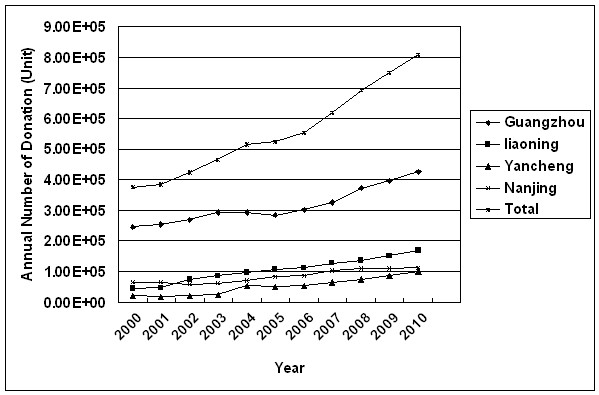
Changes in amount of WB collections among four Chinese blood centers between 2000 and 2010

The change in whole blood collection over the period of study at the four blood centers is shown in Figure 2. Compared to 2000, the overall amount of whole blood collection in 2010 increased by 116% at a rate of 8% per year, from 374,963 units in 2000 to 808,417 units in 2010. The results of this experiment exhibited the similar increasing trend in blood collection at four blood centers.

### Demographic characteristics of the blood donors

A overall of 4,366,283 whole blood donations were collected at the four blood centers between January 1, 2000 and December 31, 2010. Demographic characteristics associated with all donations within each center are shown in Table 2. Across all centers, 59.7% of the donations came from male donors. Approximately half of the donors were 25 years old or younger. The distribution of marital status varied by centers, donors with the single marital status contributed the majority of donations in Guangzhou, but the distribution presented a majority of married donors in Yancheng. Not all blood centers collected information on marital status, occupation and education during the whole eleven years; those data were therefore excluded from subsequent analyses. Furthermore, there was no effect of education level and occupation on blood donations among Chinese donors. Approximately 60% of donations came from first-time donors and 40% from repeated donors. This trend was similar in all centers except Yancheng, where 64.7% of donations came from repeated donors. Of all donations, 53.4% were collected at collection vehicles, but 93.6% of donations at the Yancheng blood center were collected at mobile blood collection vehicles and a very small proportion of the overall donations were collected at the blood center. Overall, the percentage of donations collected at collection vehicles increased dramatically in recent years (Data not shown) in most centers.

**Table 2:** Demographic characteristics associated with all donations by center between 2000 and 2010

	**Liaoning**	**Guangzhou**	**Nanjing**	**Yancheng**	**Overall**
**Number of donors**	774570	2466834	683704	442259	4366283
**Male**	460052 (59.5)	1510573 (61.2)	379270 (55.5)	258048 (58.3)	2605938 (59.7)
**Female**	314118 (40.5)	956261 (38.8)	304434 (44.5)	184211 (41.7)	1759955 (40.3)
**Age group(years)**					
**18- < 25**	355140 (45.9)	1329575 (53.9)	417408 (61.1)	52600 (11.9)	2154555 (49.4)
**25- < 35**	200156 (25.9)	706815 (28.7)	145686 (21.2)	105191 (23.8)	1157829 (26.5)
**35- < 45**	157551 (20.3)	325483 (13.2)	87383 (12.8)	142688 (32.2)	713108 (16.3)
**45- < 55**	59778 (7.8)	104961 (4.2)	33224 (4.9)	141780 (32.1)	340451 (7.8)
**Marriage status**					
**Single**	388339 (50.2)	1680942 (68.1)	*175809 (25.7)	124645 (28.2)	2369735 (54.3)
**Married**	364818 (47.0)	769425 (31.2)	*69534 (10.2)	256732 (58.1)	1460509 (33.4)
**Missing**	21413 (2.8)	16467 (0.7)	418361 (61.2)	60882 (13.7)	517123 (11.8)
**Occupation**					
**Student**	358804 (46.3)	510756 (20.7)	148330 (21.7)	33718 (7.6)	1051608 (24.1)
**Employed,self-employed, retired**	246006 (31.8)	880990 (35.7)	289239 (42.3)	151864 (34.3)	1568099 (35.9)
**Other**	168676 (21.8)	1065088 (43.2)	18571 (2.7)	249817 (56.5)	1502152 (34.4)
**Missing**	1084 (0.1)	10000 (0.4)	227564 (33.3)	6860 (1.6)	245508 (5.6)
**Education**					
**Below high school**	139787 (18.1)	615813 (25.0)	*17944 (5.5)	31869 (7.2)	805413 (20.1)
**High school**	108660 (14.1)	585030 (23.7)	*19618 (6.0)	107847 (24.4)	821155 (20.5)
**Some college**	130250 (16.8)	543266 (22.0)	*59781 (18.4)	119207 (27.0)	852504 (21.3)
**College degree and above**	101880 (13.2)	722725 (29.3)	*92389 (28.4)	69295 (15.7)	986289 (24.6)
**Other**	292909 (37.8)		135353 (41.6)	114040 (25.7)	542302 (13.5)
**Donor status**					
**first-time donor**	459551 (59.3)	1665940 (67.5)	482211 (70.5)	156010 (35.3)	2763712 (63.3)
**Repeat**	313935 (40.7)	800894 (32.5)	201493 (29.5)	286249 (64.7)	1602571 (36.7)
**Donation site: collection vehicle**	426683 (55.1)	1020364 (41.4)	472788 (69.2)	413801 (93.6)	2333636 (53.4)

Data are shown as number (%).

* these data was from 2006–2010.

### Overall seroprevalence and trend of HIV, HBV, HCV and syphilis among blood donors

The serologic prevalence status of the 4,366,283 donations examined with dual-ELISA tests for HIV, HBV, HCV and syphilis in 2000 through 2010 by the four centers is presented in Table 3. The overall seroprevalence rates of HIV, HBV, HCV and syphilis were 0.08%, 0.86%, 0.51% and 0.47%, respectively.

**Table 3:** Prevalence of serologic markers for TTI by dual ELISA-reactive status at four Chinese blood centers in 2000 through 2010

**Blood center**	**2000**	**2001**	**2002**	**2003**	**2004**	**2005**	**2006**	**2007**	**2008**	**2009**	**2010**	**Overall**
**HBV**												
Yancheng	0.47	0.49	0.35	0.23	0.58	0.42	0.37	0.38	0.41	0.27	0.20	0.37§
Nanjing	0.70	0.78	0.57	0.57	0.46	0.47	0.44	0.38	0.40	0.38	0.36	0.49§
Guangzhou	0.85	1.23	1.05	0.95	1.01	1.07	0.92	0.72	0.81	0.88	0.90	0.93§
Liaoning	4.97	3.35	1.58	1.28	0.86	0.56	0.52	0.87	1.11	0.37	0.30	1.18§
Overall	2.09	1.58	1.21	1.07	0.80	0.68	0.66	0.79	0.85	0.55	0.51	0.86
**HCV**												
Yancheng	0.94	0.47	0.44	0.26	0.33	0.34	0.25	0.17	0.15	0.24	0.24	0.29§
Nanjing	0.84	0.45	0.38	0.32	0.33	0.47	0.20	0.20	0.23	0.31	0.32	0.36§
Guangzhou	0.34	0.62	0.55	0.33	0.38	0.23	0.26	0.24	0.46	0.58	0.71	0.47§
Liaoning	2.29	1.85	1.41	1.16	1.04	0.52	0.53	0.63	0.65	1.05	0.72	0.99§
Overall	0.76	0.75	0.70	0.50	0.47	0.33	0.29	0.30	0.42	0.59	0.56	0.51
**HIV**												
Yancheng	0.32	0.36	0.13	0.10	0.33	0.28	0.11	0.16	0.13	0.10	0.11	0.18§
Nanjing	0.02	0.01	0.00	0.00	0.00	0.13	0.10	0.08	0.16	0.12	0.17	0.08
Guangzhou	0.00	0.01	0.02	0.02	0.02	0.02	0.02	0.02	0.02	0.02	0.02	0.02§
Liaoning	0.69	0.25	0.11	0.20	0.23	0.13	0.15	0.08	0.22	0.31	0.25	0.22§
Overall	0.13	0.06	0.04	0.06	0.09	0.08	0.06	0.06	0.09	0.10	0.10	0.08
**Syphilis**												
Yancheng	0.65	0.75	0.48	0.83	0.88	0.75	0.63	0.60	0.69	0.69	0.70	0.70§
Nanjing	0.30	0.36	0.37	0.40	0.36	0.47	0.35	0.25	0.33	0.30	0.44	0.36§
Guangzhou	0.21	0.29	0.34	0.25	0.30	0.36	0.50	0.53	0.46	0.47	0.66	0.42§
Liaoning	1.00	1.04	1.02	0.71	0.43	0.60	0.57	0.50	0.41	0.38	0.47	0.60§
Overall	0.37	0.44	0.49	0.41	0.38	.046	0.50	0.48	0.45	0.46	0.63	0.47

Data are reported as percentages. § p < 0.01, the p value relates to the overall prevalence.

The overall seroprevalence of HBsAg was estimated to be 0.37%, 0.49%, 0.93% and 1.18% at Yancheng, Nanjing, Guangzhou and Liaoning, respectively. Each of the center-specific seroprevalences differed significantly from the overall mean seroprevalence. Although the prevalence in Guangzhou displayed a steady tendency to HBV prevalence, there was a significant decline over these eleven years across other three blood centers. With a similar relative order as for HBsAg mean seroprevalence and the prevalence trend, all four blood centers demonstrated statistical differences in the seroprevalence of HCV except in Guangzhou which was again relatively stable.

The results from the four blood centers indicated significantly distinct HIV prevalence rates as well. The lowest positivity for HIV (0.02%) appeared at the Guangzhou blood center, which the rate was four to ten times lower than those in the other three blood centers. Although HIV prevalence in Nanjing was also relatively low (0.08%), it exhibited an upward annual trend. During 2000 and 2001, the prevalence of HIV displayed a relatively high level among the blood donors in Yancheng and Liaoning, but the data showed a decreasing and relatively lower value in the remaining years of the study.

The prevalence of syphilis also varied among these blood centers. In Nanjing, the prevalence of syphilis was lowest (0.36%), with a relatively stable rate. By contrast, the prevalence in Yancheng was highest (0.70%), with a stable rate. Guangzhou is one of the largest Chinese blood centers and revealed a gradual growth trend in syphilis prevalence. Liaoning indicated a downward trend of syphilis prevalence over the period.

In addition to the differences by year, the overall serologic positivity rate of these four pathogens also varied by donors’ age, gender, educational level, marital status, occupation and donor status (Table 4). At all four blood centers, donors under the age of 25 displayed significant lower positivity for HBV and syphilis. The prevalence of syphilis was two to four times lower in this group than in the older age groups. As for HCV, donors above the age of 46 demonstrated the lowest positivity rate at all four blood centers; this rate was significantly lower than that of the other age groups except among donors from the Guangzhou blood center. As for HIV, no significant difference was found in the four age groups except among donors from the Nanjing blood center, where the oldest age group (46–55 years old) displayed a significantly lower positivity rate. A higher positivity rate for HBV was associated with male at all four blood centers and male was highly susceptible to other three pathogens in the Guangzhou blood center as well. However, in Yancheng, females were correlated with a higher positivity rate of HIV, HCV and syphilis. There was a close correlation between higher positivity rates and lower education levels, especially for syphilis. The positivity rate of syphilis among donors who had completed a university degree or more educational levels was one-fifth to one-half the rate among those with a high school degree or less education. Donors who identified themselves as students or single marital status displayed a lower positivity rate than those who were employed or married. The data reflect that the prevalence rate of HBV and syphilis was correlated with working status in all four blood centers. Donations made by first-time donors had 50-100% higher TTI screening prevalence in HBsAg, HCV, HIV and syphilis than donations made by repeated donors except in donations from the Yancheng blood center where TTI prevalence was almost identical between the two groups.

**Table 4:** Prevalence of serologic markers for TTIs by blood center and demographic characteristics

	**Yancheng**				**Nanjing**				**Guangzhou**				**Liaoning**			
**Donor characteristics**	**HIV**	**HBV**	**HCV**	**Syphilis**	**HIV**	**HBV**	**HCV**	**Syphilis**	**HIV**	**HBV**	**HCV**	**Syphilis**	**HIV**	**HBV**	**HCV**	**Syphilis**
**Age (years)**																
18-25	0.15	0.28	0.35	0.29	0.08	0.44	0.37	0.16	0.00	0.69	0.39	0.19	0.24	1.21	0.98	0.33
26-35	0.18	0.37	0.27	0.58	0.07	0.57	0.38	0.64	0.01	0.94	0.37	0.28	0.21	1.24	0.96	0.74
36-45	0.21	0.43	0.34	0.84	0.08	0.52	0.32	0.66	0.02	1.07	0.48	0.71	0.19	1.27	1.03	0.93
46-55	0.16	0.33	0.24	0.80	0.04	0.55	0.22	0.59	0.01	0.93	0.52	0.75	0.20	1.04	1.00	0.83
**Sex**																
male	0.17	0.42	0.27	0.63	0.08	0.57	0.38	0.34	0.02	1.23	0.51	0.49	0.23	1.29	0.99	0.60
female	0.19	0.30	0.32	0.80	0.07	0.38	0.33	0.37	0.01	0.52	0.28	0.29	0.22	1.03	0.99	0.61
**Education**																
Middle school or less	0.09	0.21	0.24	0.10	0.13	0.60	0.25	0.74	0.02	1.32	0.62	0.69	0.19	0.67	0.65	0.56
High school graduate	0.15	0.67	0.43	0.75	0.13	0.54	0.24	0.83	0.01	0.91	0.46	0.39	0.18	0.67	0.68	0.54
Technician certificate	0.03	0.25	0.21	0.56	0.18	0.47	0.25	0.56	NA	NA	NA	NA	0.20	0.54	0.67	0.37
Associate degree	0.10	0.43	0.35	0.54	0.13	0.31	0.28	0.22	0.003	0.68	0.47	0.27	0.19	0.55	0.64	0.38
Complete university and above	0.09	0.22	0.21	0.34	0.12	0.28	0.31	0.11	0.01	0.82	0.31	0.18	0.19	0.54	0.74	0.26
**Marital**																
Single	0.18	0.37	0.29	0.69	0.13	0.33	0.30	0.19	0.01	0.20	0.17	0.18	0.20	1.04	0.85	0.53
Married	0.18	0.38	0.31	0.71	0.15	0.52	0.22	0.70	0.02	1.28	0.55	0.52	0.25	1.29	1.14	0.68
**Occupation**																
Student	0.17	0.24	0.37	0.16	0.10	0.30	0.33	0.13	0.007	0.87	0.34	0.09	0.26	0.28	1.03	0.21
Working(employed)	0.17	0.37	0.28	0.74	0.12	0.45	0.26	0.45	0.01	0.92	0.43	0.48	0.16	0.48	0.49	0.34
**Donor status**																
First time	0.18	0.37	0.32	0.71	0.09	0.61	0.44	0.42	0.02	1.3	0.50	0.52	0.25	1.46	1.08	0.69
Repeated	0.18	0.37	0.29	0.70	0.05	0.25	0.19	0.23	0.01	0.47	0.33	0.28	0.19	0.67	0.83	0.45

Data are reported as percentages.

## Discussion

China is facing critical challenges in blood availability and safety. Although the volume of blood collected for transfusion has constantly increased at a rate of 8% per year in China (Figure 1), the blood supply has not kept pace with the clinical demand, so China faces consistent seasonal blood shortage [14]. Despite the successful shift from paid and employer-organized donations to voluntary donations in recent years, the emerging HIV epidemic, the growing syphilis epidemic and the high HBV and HCV prevalence in the general population [7-10] are still considered serious blood safety problems in China. To ensure blood safety, it is critical to monitor ongoing epidemiological information, not only from high-risk groups, but also from individuals from the general population such as volunteer blood donors. The donor demographic characteristics of these four blood centers are quite different from the donor profile of many Western countries. For example, 60% of donors were male, half were under age 25, most donors in Guangzhou were single, and 60% of donations were from first-time donors (Table 2). To ensure an adequate blood supply, it is critical to recruit suitable blood donors. In China, the recruiting strategies to encourage the current 60% of first-time donors to return for regular donation are very important to compensate for the blood shortage.

As the number of HIV cases continues to grow in China, transfusion-transmissible HIV infection poses an increasing threat to blood safety. By the end of August 2010, there were 361,599 overall reported HIV positives, including 127,203 AIDS cases and 65,104 recorded deaths [15]. The Chinese CDC report estimated that for people living with HIV(about 650,000) by the end of 2005 in China, about 10.7% of all HIV infections in China [16] were infected by receiving blood or blood products (approximately totaling 69,550 cases). The majority of these cases were infected in late 1980’ and early 1990’ as a result of illegal plasma and whole blood collection practices[17]. The Blood Donation Law published in 1998 and the closing down of illegal plasma and whole blood collection operations by the government have greatly reduced the cases of transfusion transmitted HIV infections in China after 1998 but there is no official published data on the actual cases since 2005. One study stated that the prevalence rate of HIV infection was about 0.085% among donors from Zhejiang Province, with a growing frequency from 1:600,000 in 1995 to 1:37,500 in 2004[18]. Another meta-analysis indicated the prevalence and trend of HIV infection among voluntary blood donors in China since implementation of the Blood Donation Law (1998) [19]. This report demonstrated that an overall 2573 HIV positive cases were identified among voluntary blood donors between 2000 and 2009 in twenty-nine provinces with a prevalence of 13.33/100 000 and the overall prevalence increasing steadily and quickly. In the present work, the results demonstrated a declining trend of HIV prevalence between 2000 and 2002, and exhibited a mild fluctuation with a slightly increasing trend in later years (Table 3). Our results showed varying HIV prevalence rates among different regions in China, Liaoning (0.22%) and Yancheng (0.18%) displayed four to ten times higher HIV prevalence among blood donors than Guangzhou (0.02%) and Nanjing (0.08%).

Since the nationwide HBV vaccination program was implemented in 1992, the prevalence of HBsAg in the Chinese population has dropped to 7.2%, but China still has high HBV prevalence, with approximately 100 million individuals with chronic HBV [8]. Due to pre-donation HBsAg rapid testing, the prevalence of HBsAg (0.86%) was much lower in the donor population than that in the general population. Furthermore, present data also indicated a significant reduction in HBsAg contamination in the blood supply over the 11-year study period despite varying by blood center, such as in Guangzhou, where there is a demonstrated steady prevalent trend (Table 3). Similar decline has been reported in developed and developing countries [20-23]. The seroprevalence of HBV was significantly higher among donors who were in the higher age groups, male, less educated, employed, married or first-time donors, as compared to those who were among the lower age groups, female, more educated, students, single or repeated donors, respectively (Table 4).

Since the Blood Donation Law was enacted in 1998, the prevalence of HCV infection has been reduced dramatically in the general population. Two reasons may contribute to the significantly higher prevalence in blood donors than that in the general population before the Blood Donation Law was enacted. Firstly, before 1998, due to paid blood donations, blood centers pooled and centrifuged the blood; they retained the plasma and re-infused donors with red blood cells from the pool, which enabled people to sell their plasma more frequently without developing anemia. Secondly, this may reflect a significant testing bias and perhaps inaccessibility of anti-HCV testing among the general population, which might attract a higher proportion of high-risk persons to donate blood for the purpose of determining their infection status. However, the prevalence of HCV infection slumped dramatically to 1.71% after 1998 [9]. As presented in Table 3, the prevalence of HCV in China was significantly lower than it had been before 1998, with distinguishable differences among various regions. From 2000 to 2006, the prevalence rate showed a significant decreasing trend. After 2006, the rate started to increase probably due to a widespread HCV epidemic. Previous data have shown a high prevalence rate of HBV and HCV in China, and both viruses share several common infectious routes. Both viruses displayed a significant decline among blood donors over the study period. This decline may imply a decline in HBV and HCV in the segment of the population recruited to donate; a continuing increase in the proportion of the population that has been diagnosed with both viruses; or improved effectiveness of education and screening processes, resulting in the deferral of persons reporting high-risk behaviors. As previously discussed, lower prevalence rates for HBV (0.86%) and HCV (0.51%) were detected in blood donors than in the general population. This difference implies that predonation screening and/or self-selection of blood donors is effective.

China has experienced a dramatic resurgence of syphilis during the last two decades. Since sexually transmitted disease (STD) studies in China have reported syphilis prevalence to be about 10 times higher than HIV prevalence in both high-risk and low-risk populations, it is considered the most pressing public health issues[24]. One study also suggested there was a significant growth in syphilis serologic markers among first-time donors, with 0.41%, 0.45%, and 0.57% positivity from 2008 to 2010[13]. Our data indicated the complicated prevalence of syphilis during the study periods. As indicated in Table 3, Yancheng and Nanjing displayed a relatively constant high level of syphilis prevalence over the period; Guangzhou exhibited a continually growth trend from 0.21% in 2000 to 0.66% in 2010; and Liaoning demonstrated a continually decreasing trend from 1.00% in 2000 to 0.47% in 2010. Higher prevalence rates were also associated with donors who were older than 25 years, less educated, married, employed, or first-time donors (Table 4). The overall prevalence rate was high although the data varied among different regions, time and characteristic donors. Low education level, high age, marriage, employment and first-time donation should be the risk factors for syphilis infection.

Before 1998, TTIs were very common in China due to contaminated blood collection, particularly in for-profit plasma collection facilities. To eliminate this risk, the Chinese government has implemented many strategies, including banning paid blood donations and recruiting certain employer-organized and volunteer donations among low-risk populations [25]. The government has also implemented more sensitive screen reagents and different screening strategies. In recent years, the proportion of volunteer donors has rapidly increased, from 5% in 1998 to 71.5% at the end of 2004 and to 99% at 2011. They now constitute the main source of the blood supply in all major blood banks in China [26].

There are some limitations of this study. Firstly, we did not do confirmatory tests using widely accepted assays to perform further testing on these pathogens reactive and inconclusive donations. The prevalence of these pathogens in blood donors could be over-evaluated, potentially affecting our findings about both donor blood-borne pathogen prevalence and the relative prevalence of these pathogens between donors and the general population. Secondly, we have not analyzed these donors TTIs prevalence in a way to estimate the TTIs residual risk. Thirdly, not all centers used the same kits during the study period, and the assays varied among four blood centers, also weakening the conclusions about the regional differences in prevalence.

In the present study, we investigated the overall prevalence of various TTIs in blood donors giving blood at four regional Chinese blood centers from 2000 to 2010. The overall prevalence rates for HIV, HBV, HCV and syphilis were 0.08%, 0.86%, 0.51% and 0.47%, respectively. The seroprevalence of TTIs appears to be substantially lower among blood donors than in the general population of China, because of successful screening and the selection of donors who are at a lower risk for infection. Although the risk of TTI has been reduced dramatically, the majority of Chinese blood centers currently still rely on serology tests, with no NAT testing for routine donor screening. A few studies have demonstrated a relatively high residual risk compared to developed countries [27-30]. Fortunately, the government has realized the importance of NAT testing. In 2011, NAT testing on HBV, HCV, and HIV was piloted in eleven selected blood centers. It will be implemented at all provincial-level blood centers in the next several years. The population prevalence of TTIs which potentially threaten the safety of the blood supply, along with rising transfusion demands for blood, have stimulated an increased awareness of the importance of strengthening the safety of the blood supply and blood transfusion in China. We believe the residual risk of TTIs will be greatly reduced after NAT implementation.

## Conclusions

The decreased prevalence rate of HBV and HCV in Chinese blood donors suggests that the persistent benefit of screening strategies undoubtedly exists. However, with a higher population prevalence of certain transfusion transmissible blood borne pathogens, and until widespread implementation of more sensitive donor screening (such as nucleic acid testing), the residual risk of many TTIs is most likely higher and to remain so, than in developed countries. Additional work, particularly in the implementation of NAT testing, needs to be done to ensure blood safety and availability in China.

## Abbreviations

TTIs: Transfusion transmissible infections; NAT: Nucleic acid amplification test; HBsAg: Hepatitis B virus surface antigen; EIA: Enzyme immunoassay; ALT: Alanine aminotransferase.

## Competing interests

The authors declare that they have no competing interests.

## Authors’ contributions

WL designed, searched data and literature and gave a critical view of manuscript writing. HY gave critical view of manuscript writing. All the other authors collected and analyzed the data. All the authors’ read and approved the final manuscript.

## Author information

Li Changqing and Xiao Xiaopu are Joint first author.
